# Thoracic trauma severity contributes to differences in intensive care therapy and mortality of severely injured patients: analysis based on the TraumaRegister DGU®

**DOI:** 10.1186/s13017-017-0154-1

**Published:** 2017-09-02

**Authors:** Jörg Bayer, Rolf Lefering, Sylvia Reinhardt, Jan Kühle, Jörn Zwingmann, Norbert P. Südkamp, Thorsten Hammer, TraumaRegister DGU

**Affiliations:** 1 Department of Orthopedics and Trauma Surgery, Medical Center – Albert-Ludwigs-University of Freiburg, Faculty of Medicine, Albert-Ludwigs-University of Freiburg, Hugstetter Str. 55, 79106 Freiburg, Germany; 2 FOM - Institute for Research in Operative Medicine, University Witten/Herdecke, Faculty of Health, Ostmerheimer Str. 200, 51109 Köln, Germany; 3 Department of Orthopedics and Trauma Surgery, Oberschwabenklinik St. Elisabeth, Elisabethenstr. 15, 88212 Ravensburg, Germany; 4 Committee on Emergency Medicine, Intensive Care and Trauma Management of the German Trauma Society (Sektion NIS), Berlin, Germany

**Keywords:** Severely injured, Polytrauma, Thoracic trauma, Chest injury, Organ failure, Mortality

## Abstract

**Background:**

Thoracic trauma is a relevant source of comorbidity throughout multiply-injured patient care. We aim to determine a measurable influence of chest trauma’s severity on early resuscitation, intensive care therapy, and mortality in severely injured patients.

**Methods:**

Patients documented between 2002 and 2012 in the TraumaRegister DGU®, aged ≥ 16 years, injury severity score (ISS) ≥ 16 are analyzed. Isolated brain injury and severe head injury led to exclusion. Subgroups are formed using the Abbreviated Injury Scale_Thorax_.

**Results:**

Twenty-two thousand five hundred sixty-five patients were predominantly male (74%) with mean age of 45.7 years (SD 19.3), blunt trauma (95%), mean ISS 25.6 (SD 9.6). Overall mean intubation period was 5.6 days (SD 10.7). Surviving patients were discharged from the ICU after a mean of about 5 days following extubation. Thoracic trauma severity (AIS_Thorax_ ≥ 4) and fractures to the thoracic cage significantly prolonged the ventilation period. Additionally, fractures extended the ICU stay significantly. Suffering from more than one thoracic injury was associated with a mean of 1–2 days longer intubation period and longer ICU stay. Highest rates of sepsis, respiratory, and multiple organ failure occurred in patients with critical compared to lesser thoracic trauma severity.

**Conclusion:**

Thoracic trauma severity in multiply-injured patients has a measurable impact on rates of respiratory and multiple organ failure, sepsis, mortality, time of mechanical ventilation, and ICU stay.

## Background

Chest trauma ranks as the most important injury in severely injured patients, and about 50% of those with multiple trauma suffer from an associated chest injury [1, 2]. Injury to the thorax may affect the thoracic wall (e.g., rib, sternum fracture) as well as thoracic organs (e.g., lung, heart, vessels) to a different extent. Only a minority of patients with thoracic trauma tends to develop respiratory failure requiring intubation and ventilator support to correct hypoxia and hypercapnia [3]. On the other hand, 35 to 58% of severely injured patients require prehospital intubation, depending on the severity of concomitant chest injury [4, 5]. While severely injured patients usually require intensive care therapy irrespective of accompanying thoracic injuries, organ dysfunction and multiple organ failure (MOF) are known to develop more often in patients with severe thoracic trauma [6]. In severely injured patients, including those with severe traumatic brain injury (TBI), at least one organ failed in about 52%, with lung failure occurring in 26% [6]. Overall, MOF rates of 13 to 33% have been reported in severely injured patients. While lung failure rates ranged from 50 to 65% in MOF patients [6, 7], only 7% of patients without MOF suffered from lung failure [6]. Overall, MOF patients require prolonged ventilation and remain longer in the intensive care unit (ICU), thus consuming considerable health care resources [7].

Severe multiple trauma is often associated with traumatic lung injury and presents with a wide spectrum of severity [8]; the reported mortality of chest trauma can be as high as 60% [2], and 20 to 25% of deaths in severely injured patients are attributed to chest injury [9, 10].

Taking care of a severely injured patient is demanding. Differences in prehospital and early clinical trauma management have been reported that depends on the severity of an accompanying chest trauma [5]. Multiply-injured patients with blunt chest trauma require significantly longer periods of mechanical ventilation and a significantly longer stay in the ICU than trauma patients without a thoracic injury [2]. Furthermore, chest injuries predispose to pneumonia [11], adult respiratory distress syndrome (ARDS) [12], and multiple organ dysfunction syndrome (MODS) [6]. Therefore, we aimed to determine whether the chest trauma’s severity in severely injured patients reveals a measurable influence on early resuscitation, intensive care therapy, and mortality.

## Methods

### The TraumaRegister DGU®

The TraumaRegister DGU® (TR-DGU) of the German Trauma Society (Deutsche Gesellschaft für Unfallchirurgie, DGU) was founded in 1993. The aim of this multicenter database is the anonymized and standardized documentation of severely injured patients’ care.

Data are collected prospectively in four consecutive time periods from the accident site until hospital discharge: (A) pre-hospital phase, (B) emergency room and initial surgery, (C) ICU, and (D) discharge. Documentation includes detailed information on demographics, injury pattern, comorbidities, pre- and in-hospital management, course in the ICU, relevant laboratory findings including each individual’s data on transfusions, and outcome. The inclusion criterion is admission to the hospital via the emergency room with subsequent ICU/ICM care or reaching the hospital with vital signs and dying before admission to the ICU.

The infrastructure for documentation, data management, and data analysis is provided by AUC—Academy for Trauma Surgery (AUC—Akademie der Unfallchirurgie GmbH), a company affiliated with the German Trauma Society. Scientific leadership is provided by the Committee on Emergency Medicine, Intensive Care and Trauma Management (Sektion NIS) of the German Trauma Society. The participating hospitals submit their anonymized data into a central database via a web-based application. The quality of the scientific data analysis is monitored by peer review procedure established by Sektion NIS.

The participating hospitals are primarily located in Germany (90%), but a rising number of hospitals in other countries are contributing data as well (at the moment from Austria, Belgium, China, Finland, Luxemburg, Slovenia, Switzerland, The Netherlands, and the United Arab Emirates). Currently, approximately 35,000 cases from more than 600 hospitals have been entered into the database per year.

Participation in TR-DGU is voluntary. For hospitals associated with TraumaNetzwerk DGU®, however, the entry of at least a basic data set is obligatory for reasons of quality assurance. Hospitals interested in trauma research must enter a standard data collection form that contains more comprehensive information (e.g., organ failure, sepsis) on the patient course compared with the basic data set.

### Patients

Patients documented between 2002 and 2012 in the TR-DGU were analyzed for eligibility in this investigation. Patient selection was carried out based upon the following criteria:

(1) online documentation from European trauma centers, (2) age ≥ 16 years, (3) ISS ≥ 16, (4) exclusion of isolated brain injuries, and (5) exclusion of severe head injury defined as AIS_Head_ ≥ 4 to prevent confounding, since severe head trauma may pose an indication for intubation itself [13].

Injuries were graded according to the 2005 version of the Abbreviated Injury Scale (AIS) [14], and the injury severity score (ISS) was calculated as described [15]. While the ISS is calculated from the three worst affected body regions as the sum of squares of the respective AIS severity levels, the New ISS (NISS) is calculated in a similar way, but instead of their location, the three worst injuries enter the equation [15, 16].

Length of mechanical ventilation in ICU was defined as the number of days spent in the ICU with endotracheal intubation or a tracheostomy and mandatory mechanical ventilation (excluding, e.g., non-invasive ventilation).

Length of hospital stay was defined as the time spent in primary care in the hospital participating in the TR-DGU.

Organ failure was assessed by the SOFA score as described previously. The SOFA score describes organ function in the respiratory, cardiovascular, renal, hematologic, hepatic, and central nervous systems [17]. Each organ system was graded to evaluate the severity of organ dysfunction or failure. Patients with organ failure entered in the TR-DGU database had to have met the SOFA score criteria for organ failure for at least 2 days. Multiple organ failure (MOF) and sepsis were assessed according to published guidelines [18]. Sepsis was defined as a systemic response to infection (presence of microorganisms). Data on respiratory failure, MOF, and sepsis were available on patients documented via the standard data collection form only.

Patient subgroups were defined according to the chest injury severity (AIS_Thorax_). The first group consisted of patients with no relevant thoracic injuries (AIS_Thorax_ = 0 or 1), serving as a control group (“controls”). Group AIS-2 consisted of patients with AIS_Thorax_ = 2. Group AIS-3 consisted of patients with AIS_Thorax_ = 3. Group AIS-4 consisted of patients with AIS_Thorax_ = 4. Group AIS-5/6 consisted of patients with AIS_Thorax_ = 5 and 6, comprising the highest severity of chest trauma.

AIS_Thorax_ included all thoracic injuries coded as AIS = 4xxxxx.x [14]. Injuries to the thoracic spine were excluded, as these were coded with an AIS = 6xxxxx.x number.

### Statistical analysis

Demographic and clinical characteristics comparing the aforementioned groups were evaluated using descriptive statistics. Continuous variables are presented as mean with standard deviation (SD), while categorical variables are presented as number of cases with percentages. The respective statistics refer to patients with valid data sets only. Therefore, the total number of patients or characteristics may sometimes vary.

Data for respiratory failure (ARDS), sepsis, and MOF are not part of the basic data set; therefore, not all hospitals provide this information, and patient numbers vary.

Formal statistical testing would require an initial overall test (chi-squared, analysis of variance, or Kolmogoroff-Smirnov) followed by pair-wise comparisons in case of significance. With five subgroups, the number of pair-wise tests would be ten per variable. Since the number of patients in the five subgroups ranged from 2000 to 8000, even minor differences would reach statistical significance. The 95% confidence interval in groups of 2000 cases (or more) would be about +/− 2% (or less) in case of categorical variables, and +/− 0.025*SD in case of continuous variables. For these reasons, we refrained from formal statistical testing, and our analysis is mainly descriptive.

Statistical testing for influencing factors on length of ICU stay and mechanical ventilation was performed using multiple linear regression analysis. In this analysis, the dependent variables were length of ICU stay and length of mechanical ventilation, respectively. The independent variables consisted of patient characteristics (age ≥ 60 years, gender, concomitant diseases), injury severity (ISS, AIS_Thorax_ ≥ 4, AIS_Head_ = 3, AIS_Abdomen_ ≥ 3, AIS_Extremity_ ≥ 3), therapeutic interventions (ICU admission intubated/ventilated, blood transfusion), and type of injury to thoracic structures (vascular, lung, bone). Only surviving patients were included in this statistics. A *p* value of < .05 was considered significant.

All data were analyzed using SPSS, version 22.0 (IBM Inc., Armonk, NY, USA).

To analyze changes over time, we divided our 10-year data set into subgroups of 2 consecutive years each.

The present study is in line with the publication guidelines of the TraumaRegister DGU® and registered as TR-DGU project ID 2011–015.

Our study cohort has been described, analyzed for different characteristics, and published before [5].

## Results

Data of 37,495 severely injured patients, including patients with head injuries of any severity, with an ISS ≥ 16 and aged ≥ 16 years were entered into the database. Of these, 64.3% suffered from at least moderate (AIS_Thorax_ ≥ 2) and 26.7% from severe thoracic injury (AIS_Thorax_ ≥ 4), respectively.

After excluding patients suffering from severe head injury (AIS_Head_ ≥ 4), a total of 22,565 severely injured patients aged a mean 45.7 years (SD 19.3) and presented a mean ISS of 25.6 (SD 9.6) were identified for further analysis.

The standard data collection form (including data for respiratory failure, MOF, and sepsis) was filed for 16,793 (74.4%) patients, yet ~ 13% of these datasets were incomplete and excluded for analysis of the respective absent parameter.

### Demographics

The study group’s basic characteristics are summarized in Table 1. The patients suffering mainly from blunt trauma included in our study are predominantly male.

**Table 1:** Basic characteristics: groups according to the AIS_Thorax_ (0 + 1, 2, 3, 4, and 5 + 6)

	Controls *n* = 4870	AIS-2 *n* = 1973	AIS-3 *n* = 8052	AIS-4 *n* = 5433	AIS-5/6 *n* = 2237
Age (years) Mean ± SD	44.2 ± 19.6	41.7 ± 18.6	45.3 ± 19.2	47.4 ± 19.0	49.3 ± 19.1
ISS Mean ± SD	21.5 ± 6.4	21.4 ± 5.9	23.5 ± 6.9	28.1 ± 9.3	39.4 ± 12.5
New ISS Mean ± SD	25.5 ± 8.3	23.5 ± 7.4	26.2 ± 7.4	33.7 ± 9.7	48.4 ± 12.6
Males	3439 71.3%	1414 72.0%	6009 75.1%	4074 75.5%	1667 75.3%
Blunt trauma	4405 93.7%	1855 97.0%	7518 96.5%	5028 95.7%	1979 92.0%

Total numbers and percentages for gender and injury mechanism

Total numbers for gender and trauma mechanism differ from the initial group

The distribution of injuries to different thoracic structures is dependent on thoracic trauma severity and is shown in Table 2. Most patients suffer from injuries to the lung, whereas injury to intrathoracic vessels is less prevalent in patients suffering from thoracic trauma (AIS_Thorax_ ≥ 2).

**Table 2:** Distribution of injuries to different thoracic structures in patients with at least moderate thoracic injury

	AIS-2	AIS-3	AIS-4	AIS-5/6	AIS_Thorax_ ≥ 2
Vascular	0.2%	0.5%	4%	12.7%	542 (3.1%)
Lung	68.7%	80%	91.9%	84.5%	14,640 82.7%
Bone	39.8%	55.8%	46%	65.1%	9238 52.2%

Groups according to the AIS_Thorax_ (2, 3, 4, and 5 + 6) and all patients with AIS_Thorax_ ≥ 2

Number of patients (with AIS_Thorax_ ≥ 2) and percentages are given for “vascular” (intrathoracic vascular injury), “lung” (e.g., lung contusion, laceration, pneumothorax) and “bone” (rib and sternal fractures) injuries

### Intensive Care

After primary work up in the emergency department, the rate of patients transferred directly to the Intensive Care Unit (ICU) was higher for AIS_Thorax_ ≥ 3 (42.3– 47.8%) than AIS_Thorax_ ≤ 2 (37.1–30.8%). The remaining patients underwent, for example, early (e.g., external fracture stabilization) or emergency (e.g., laparotomy) surgery.

The higher their AIS_Thorax_ score, the more patients who were admitted to the ICU were intubated and mechanically ventilated. Additionally, a one-point increase in the AIS_Thorax_ resulted in about a 1-day longer intubation period in ICU. Overall, we report a mean intubation period of 5.6 days (SD 10.7) in all our severely injured patients. Again, length of the ICU stay increased with the AIS_Thorax_ score and was a mean of 10.1 days (SD 13.4) in our overall population **(**Table 3 **)**.

**Table 3:** Influence of thoracic trauma severity on mechanical ventilation and ICU/hospital stay in all patients

	Controls	AIS-2	AIS-3	AIS-4	AIS-5/6
Mechanical ventilation/intubation	2708 (62.7%)	1199 (65.4%)	5003 (66.7%)	3525 (71.9%)	1499 (81.1%)
Length of mechanical ventilation	3.9 ± 9.9 [1]	4.6 ± 9.7 [1]	5.2 ± 9.8 [1]	7.1 ± 11.4 [2]	8.1 ± 12.9 [2]
Length of ICU stay	8.1 ± 12.7 [4]	9.3 ± 13.7 [5]	9.8 ± 12.3 [5]	11.7 ± 13.9 [7]	12.6 ± 16.4 [7]
Length of hospital stay	28.1 ± 27.2 [21]	28.4 ± 27.2 [21]	26.5 ± 24.1 [20]	25.5 ± 23.6 [20]	23.4 ± 24.2 [18]

Number of patients and percentages requiring mechanical ventilation during their ICU stay. Length of mechanical ventilation, ICU stay, and hospital stay in days (mean ± SD); [median]. Subgroups according to the AIS_Thorax_ (0 + 1, 2, 3, 4, and 5 + 6)

**Fig. 1:**
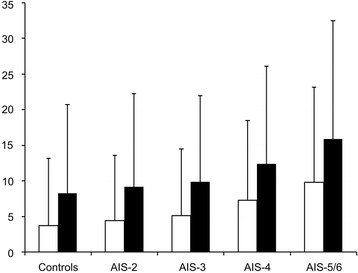
Influence of thoracic trauma severity on mechanical ventilation and ICU stay in surviving patients. Length of mechanical ventilation (open boxes) and ICU stay (black columns) in days (mean value + standard deviation) for different thoracic trauma severity. Subgroups according to the AIS_Thorax_ (0 + 1, 2, 3, 4, and 5 + 6)

In the subgroup of surviving patients only, the higher their AIS_Thorax_ score was, the longer the intubation period and ICU stay were. The mean duration of intubation was 5.6 days (SD 10.4) and mean length of ICU stay was 10.6 days (SD 13.2) for all groups presenting different thoracic trauma severity. Within each group, patients stayed in the ICU for a mean of approximately 5 days after extubation **(**Fig. 1 **)**.

In our multiple linear regression analysis for influencing factors (patient characteristics, injury severity, therapeutic interventions, and type of injury to thoracic structures) on ICU length of stay and length of mechanical ventilation, we found no effect for higher thoracic trauma severity (AIS_Thorax_ ≥ 4) on ICU length of stay (+ 0.2 days; *p* = 0.54) but a significant effect on intubation days (+ 0.7 days; *p* = 0.005).

While vascular and lung injury did not significantly affect ICU length of stay and ventilation days, fractures significantly prolonged ICU length of stay (+ 1.7 days; *p* < 0.001) and intubation days (+ 0.9 days; *p* < 0.001).

We detected an influence from having one versus several diagnosed thoracic injuries on the duration of both mechanical ventilation and length of ICU stay, namely a one- to two-day mean increase in the duration of mechanical ventilation and ICU stay in patients suffering from multiple thoracic injuries. This was seen within each group of thoracic trauma severity (AIS_Thorax_) ≥ 3. Again, irrespective of the counted thoracic injuries, patients remained about 5 days longer in the ICU than being mechanically ventilated **(**Table 4 **)**.

**Table 4:** Mechanical ventilation and length of ICU stay is dependent on the number of thoracic injuries

	AIS-2		AIS-3		AIS-4		AIS-5/6	
	One	Mul	One	Mul	One	Mul	One	Mul
Mechanical ventilation	4.7	4.2	4.7	5.8	6.4	7.5	6.2	8.6
ICU stay	9.4	8.9	8.9	10.8	10.6	12.3	9.8	13.4

Mechanical ventilation time and length of ICU stay in mean days for singular or multiple chest trauma diagnoses

Subgroups according to the AIS_Thorax_ (2, 3, 4, and 5 + 6)

In patients scoring an AIS_Thorax_ ≥ 2, one thoracic injury meant a mean 5.2 days of mechanical ventilation and 9.5 days of ICU stay versus 6.9 and 11.8 days with multiple thoracic injuries, respectively.

### Length of hospital stay

**Fig. 2:**
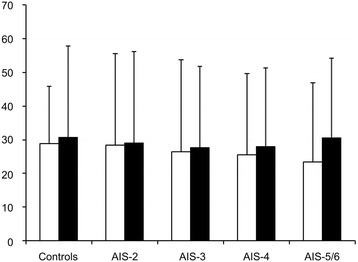
Influence of thoracic trauma severity on hospital stay in all severely injured and surviving patients. Length of hospital stay in days (mean value + standard deviation) for different thoracic trauma severity in all severely injured (open boxes) and surviving patients only (black columns). Subgroups according to the AIS_Thorax_ (0 + 1, 2, 3, 4, and 5 + 6)

The length of hospital stay of all our severely injured patients decreased from a mean of 28.1 days (SD 27.2) in the controls with an AIS_Thorax_ ≤ 1 to 23.4 days (SD 24.2) for those with AIS-5/6 **(**Table 3 **)**. When considering the surviving patients only, we identified no differences in length of hospital stay in conjunction with varying chest trauma severities **(**Fig. 2 **)**. Overall, the surviving patients, irrespective of their chest trauma severity, were hospitalized a mean of 28.8 days (SD 24.9).

While multiple chest trauma diagnoses in our severely injured patients—compared to a single diagnosis—were not associated with longer hospital stays for AIS_Thorax_ ≤ 4, patients with an AIS_Thorax_ ≥ 5 suffering from multiple chest trauma had an approximately 3.5 days longer mean hospital stay than patients presenting a single thoracic trauma diagnosis (20.9 versus 24.2 days).

### Organ failure/mortality

Our severely injured patients revealed a higher rate of lung failure (defined as a Horowitz index ≤ 200 mmHg for at least 2 days (SOFA-Score [17]) the higher their AIS_Thorax_ was. While 13.9% of the patients with an AIS_Thorax_ ≤ 1 suffered from acute lung failure, 40.3% of those with an AIS_Thorax_ ≥ 5 did.

Rates of sepsis, respiratory failure, and MOF were higher in patients with greater thoracic trauma severity. The AIS-5/6 group displayed more than twice the rate of MOF, respiratory failure, and sepsis than the control subgroup **(**Table 5 **)**.

**Table 5:** Organ failure, sepsis, and mortality rates are dependent on thoracic trauma severity

	Controls	AIS-2	AIS-3	AIS-4	AIS-5/6
Multiple organ failure^a^	625 (19.3%)	268 (20.4%)	1171 (22.5%)	1107 (30.7%)	534 (42.4%)
Respiratory failure^a^	450 (13.9%)	231 (17.6%)	1130 (21.7%)	1157 (32.1%)	506 (40.3%)
Sepsis^a^	231 (7.1%)	107 (8.2%)	477 (9.1%)	411 (11.4%)	203 (16.0%)
Early mortality	254 (5.2%)	44 (2.2%)	276 (3.4%)	436 (8.0%)	491 (21.9%)
Hospital mortality	418 (8.6%)	93 (4.7%)	561 (7.0%)	687 (12.6%)	635 (28.4%)

Number of patients and percentage within the respective cohort of thoracic trauma severity are shown

Early mortality: death within 24 h after hospital admission

^a^Parameter available only for patients documented with the standard form (*n* = 16,793; missing data in 13%); therefore, total patient numbers vary

Early death within 24 h after admission to the hospital occurred in 6.7% of all our severely injured patients, and a total of 10.6% died during their hospital stay. When examining the distribution of chest trauma severity in our deceased patients, we found higher mortality in those with an AIS_Thorax_ ≥ 4 (this applies to the early deaths as well as all deaths during hospital stay) **(**Table 5 **)**.

Death usually occurred while the patients were in the ICU. Late mortality, meaning death occurring later than 3 days after ICU discharge, occurred more often in patients with an AIS_Thorax_ ≤ 3 (about 10% of patients in each group). An AIS_Thorax_ = 4 was associated with 5.2%, an AIS_Thorax_ ≥ 5 with 3% of the late deaths.

Patients sustaining multiple chest injuries within one AIS_Thorax_ severity score did not reveal mortality rates different from those of patients with one thoracic trauma diagnosis.

### Changes over time

**Fig. 3:**
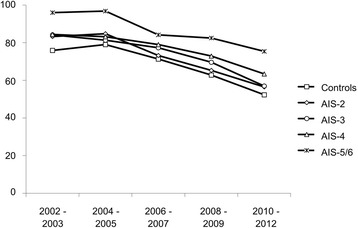
Rates of mechanical ventilation in severely injured patients changed over time. Percent of mechanically ventilated severely injured patients at ICU admission over consecutive time spans. Subgroups according to the AIS_Thorax_ (0 + 1, 2, 3, 4, and 5 + 6)

The rate of severely injured patients being admitted intubated to the ICU dropped steadily over the decade we examined. While 83% of patients arrived in the ICU on mechanical ventilation between 2002 and 2003, only 59.3% were intubated between 2010 and 2012. We noted the same pattern in all groups during those years when patients are segregated according to their chest trauma severity **(**Fig. 3 **)**.

**Fig. 4:**
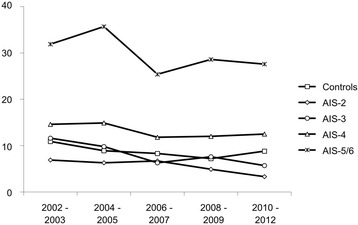
Mortality in severely injured patients changed over time. Hospital mortality rates shown over consecutive time spans for different thoracic trauma severity. Subgroups according to the AIS_Thorax_ (0 + 1, 2, 3, 4, and 5 + 6)

While the rate of acute lung failure in all groups (including the subgroups with varying chest trauma severity (data not shown)) revealed no distinct trend over the decade, mortality during hospital stay did fall from 13.5 to 10.1% for all patients from 2002 to 2012. Mortality in all groups is now lower than it was in 2002 **(**Fig. 4 **)**.

## Discussion

We present a retrospective analysis of severely injured patients suffering from thoracic traumas of different magnitude. As in other studies [6, 13, 19–22], our population consists mainly of middle-aged males with a mean ISS ≥ 16 suffering from blunt trauma. Severe head trauma (AIS_Head_ ≥ 4) alone can be an indication for intubation [13], and concomitant head and thoracic trauma influence each other in terms of intensive care therapy [21, 23]. Previous studies have shown that severely injured patients with head and chest injuries require prolonged intensive care stay and mechanical ventilation [23], and thoracic injury is known to be an independent risk factor for 30-day mortality and poor outcome in patients suffering traumatic brain injuries [21]. Thus, by excluding patients with severe head trauma (AIS_Head_ ≥ 4), we minimized the risk of confounding our target parameters by such injuries’ effects.

Severe multiple trauma is often associated with traumatic lung injury. While the prehospital intubation of patients with severe thoracic trauma without manifest respiratory insufficiency has been debated [13, 24], Ruchholtz et al. reported that 95% of their severely injured patients suffering from severe thoracic trauma (AIS_Thorax_ = 4), without prehospital intubation presented the indication for intubation over the medium term [13]. In our population, 62 to 81% of patients admitted to the ICU were already intubated, and most underwent interventions (e.g., surgery) prior to their admission. In line with others, we also detected higher rates of mechanical ventilation at ICU admission in conjunction with higher AIS_Thorax_ scores [22]. Our severely injured patients were mechanically ventilated for a mean 5.6 days—less time than Hildebrand et al.’s severely injured patients (mechanically ventilated for a mean 11 days) but comparable to Sauaia et al. Those two working groups also excluded severely injured patients with severe traumatic brain injury [7, 22]. Hildebrand et al. demonstrated a greater increase in mechanical-ventilation days in conjunction with each additional point in the AIS_Thorax_ than we did, but they only analyzed patients with an AIS_Thorax_ ≥ 3 ≤ 5 [22].

Our reported lengths of ICU stay falls within the range of Sauaia et al. and Fröhlich et al. regarding their populations of severely injured patients [6, 7]. Compared to Hildebrand et al., all our patients exhibit an almost 5-day shorter mean length of stay in the ICU, despite similar ISS scores and overall mortality, exclusion of severe head trauma in both studies, and our population’s higher percentage of acute lung failure [22].

Interesting enough, we found no statistical significant influence of more severe thoracic trauma (AIS_Thorax_ ≥ 4) on ICU length of stay, but a significant effect on prolonging the mechanical ventilation period. When breaking the AIS_Thorax_ down into actual injuries to thoracic structures, only fractures to the rib cage and sternum significantly prolonged the ICU stay and intubation. This is reflected in a scoring system where patients suffering from thoracic trauma are, among other parameters, assessed for the extent of rib fractures to estimate the requirement of mechanical ventilation and prolonged care [25].

We demonstrate with our severely injured patients that not only the most severe injury to the chest—which determines the AIS_Thorax_ score—was pertinent to the length of the intubation period, but also that the total number of chest injuries influenced both, length of mechanical ventilation and ICU stay. This may, again, be reflected in the recently developed scoring system to predict the outcome of patients with chest wall injuries where parameters like age, extent of pulmonary contusion, number of rib fractures, and existing bilateral rib fractures are taken into account. Patients with total scores ≥ 5 were mechanically ventilated and hospitalized for a longer period, while those with scores ≥ 7 revealed a still greater risk for mortality, ICU admission, and mechanical ventilation [25]. However, we detected no correlation between multiple injuries to the chest and increased mortality in our study.

The 10.6% overall mortality we report falls within the published range of in-hospital-mortality in a severely injured population (9 to 16.2%) [6, 19, 22]. To the best of our knowledge, AIS_Thorax_-dependent differences in mortality have not been reported before. Interestingly, our data reveal that single and multiple injuries resulting in an AIS_Thorax_ = 3 and 2 did not increase mortality compared to AIS_Thorax_ = 0 and 1 in our severely injured patients. Perhaps lesser injuries to the chest, despite increasing the rate of acute lung failure in our population, do not inflict greater injury to the other organ systems, and the body can recuperate. While the 10.9 to 40.3% range of AIS_Thorax_-dependent acute lung failure we report resembles the published incidence of 8 to 37% in trauma patients [26, 27], others have shown the associated mortality to range from 16 to 29% [26–28].

An AIS_Thorax_ ≥ 4 may exert widespread chest-trauma effects throughout the body, thereby furthering the development of multiple organ dysfunction and failure and raising an independent risk factor for acute lung injury (ALI), ARDS, and pulmonary failure [29, 30]. Furthermore, it is well known that 80% of patients with multiple organ dysfunction syndrome (MODS) start with lung failure [23], and that severe thoracic trauma is an independent risk factor for developing MODS [6, 31]. MODS-related deaths range from 13 to 36% [6, 7, 32]. Our data support these findings, as we noted increasing rates of respiratory failure with increasing thoracic trauma severity, and demonstrate the first major rise in MOF in association with an AIS_Thorax_ ≥ 4. In the context of sepsis, our reports are comparable to published rates in severely injured patients, which range from 3.1 to 17% [33]. However, we also demonstrate that the severity of thoracic trauma is associated with increasing sepsis rates in severely injured patients.

We report different mean LOS for all severely injured patients and surviving patients only. The mean LOS for all patients is shorter than for surviving patients and shortest for all patients with AIS-5/6. This is not surprising, since per definition AIS_Thorax_ = 6 is a not survivable injury and many patients with AIS_Thorax_ = 5 will die because of the magnitude of injury. More striking is the fact that across the AIS groups in surviving patients, LOS seems not to differ. One explanation could be that patients with lower AIS_Thorax_ exhibit more serious injuries in other body regions (e.g., abdominal, extremities) responsible for longer hospitalization in these groups. This is underlined by the fact that we only found a tendency towards longer ICU stay with increasing thoracic trauma severity, and this effect was not statistically significant in our multiple linear regression analysis. Another possibility is the early discharge of patients with higher AIS_Thorax_ for respiratory weaning/respiratory rehabilitation, thus shortening the period of care in the primary care facility documenting these cases.

When considering where death occurred, most of our patients died in the ICU. However, an AIS_Thorax_ < 4 incurred a higher rate of deaths on regular wards than an AIS_Thorax_ ≥ 4. We were unable to analyze the causes of death in these different groups since they are not documented in the TR-DGU’s primary data set. Thus, we have too little knowledge to determine why, after being discharged from the ICU, severely injured patients, without severe head and severe thoracic trauma, had a higher rate of death than patients with severe chest trauma.

The percentage of intubated, severely injured patients admitted to the ICU has declined in recent years, independent of the severity of their thoracic trauma. This might be a reflection of prehospital behavior, where changes over the last 20 years—from routine intubation in most severely injured patients to intubation based on clearer indications—has led to better care [34]. Moreover, in patients in whom the indication for mechanical ventilation arose because of the accompanying thoracic trauma, early non-invasive ventilation has proven to significantly reduce mortality and the intubation rate without increasing complications [35].

We publish a decline in the mortality rate of severely injured patients in recent years irrespective of the severity of the chest trauma they sustained. This observation concurs with prior data reporting an overall drop in the mortality of severely injured patients [6].

This study is limited by its retrospective nature. Hospitals participating in the TR-DGU are regularly audited, and sample tests are taken to ensure data quality. However, the validity of their documentation is not verified by external monitors as in prospective trials [36]. The present analysis is based upon a European population where the majority experienced blunt trauma, which might differ from cohorts with a higher percentage of penetrating injuries. Our interpretation of the causes of mortality is limited by referring to the TR-DGU database, since the actual cause of death is not recorded; thus, we could not ascribe higher mortality rates to either inherent thoracic (e.g., respiratory failure) or to other causes (e.g., sepsis, MOF). Additionally, we excluded patients with severe head injury (AIS_Head_ ≥ 4) in our study to minimize confounding and, as a result, our findings cannot be readily transferred to severely injured patients sustaining additional major head trauma.

To interpret our results, one has to keep in mind that our “control group” is mainly characterized by the absence of relevant thoracic trauma (AIS_Thorax_ ≤ 1). Thus, to generate an ISS ≥ 16 without severe injury to the head, patients needed at least one severe injury or a combination of injuries to the remaining body regions. Albeit, since we clearly focus on the influence of thoracic trauma in severely injured patients, we feel this to be an adequate “control group.”

## Conclusion

The extent of thoracic trauma in severely injured patients is a relevant risk factor for intensive care therapy, organ failure, sepsis, and mortality. We demonstrate that the higher the AIS_Thorax_ score, the higher the rate of intubated patients admitted to the ICU. Higher AIS_Thorax_ scores and multiple injuries to the chest correlated with longer periods of mechanical ventilation and ICU stays. Mortality in our population began to rise in conjunction with an AIS_Thorax_ ≥ 4. Overall, while most patient deaths occurred during their ICU stay, death later than 3 days after ICU discharge occurred more often in patients with milder chest injuries (AIS_Thorax_ ≤ 3).

## Abbreviations

AIS: Abbreviated injury scale
ALI: Acute lung injury
ARDS: Adult respiratory syndrome
ICU: Intensive care unit
ISS: Injury severity score
LOS: Length of hospital stay
MODS: Multiple organ dysfunction syndrome
MOF: Multiple organ failure
NISS: New ISS
SD: Standard deviation
TBI: Traumatic brain injury
TR-DGU: The TraumaRegister DGU®
